# Vape-Associated Pulmonary Injury (VAPI) Presenting With a “Miliary” Pattern on Imaging

**DOI:** 10.7759/cureus.13385

**Published:** 2021-02-16

**Authors:** Marcus A Threadcraft, Robert Case

**Affiliations:** 1 Internal Medicine, University of Florida College of Medicine, Gainesville, USA; 2 Internal Medicine, University of Florida Health, Gainesville, USA

**Keywords:** vape, e-cigarette, miliary, micronodular, pulmonary injury, thc, vapi

## Abstract

Electronic (E)-cigarette use or vaping is associated with pulmonary injury. Users can present with wide-ranging symptoms, varying degrees of pulmonary injury, and respiratory distress. Lung injury secondary to vaping is associated with a variety of patterns on pulmonary imaging. Typical radiographic findings are consistent with bilateral, basilar ground-glass opacities and or consolidation with septal thickening. We present a case of vape-associated pulmonary injury (VAPI) in a previously healthy adult who was found to have atypical radiographic findings. A 34-year-old male presented with a chief complaint of a two-week history of malaise, nausea, cough, and worsening shortness of breath. A chest CT scan without contrast revealed diffuse nodules in a miliary pattern. The patient reported a six-month history of tetrahydrocannabinol* *(THC) vape use. Bronchoscopy with cytologic analysis confirmed findings consistent with the VAPI. To our knowledge, this is the first report of a "miliary" pattern of infiltrates and nodules in a patient with VAPI. This pattern on CT imaging led to increased suspicion for other possible etiologies, including tuberculosis. Thus, moving forward, we believe that VAPI needs to be considered in the differential diagnosis if a patient presents with radiographic findings consistent with a miliary or diffuse micronodular pattern.

## Introduction

As an alternative to traditional cigarette smoking, electronic (E)-cigarettes or vaping has become popular among young adults and teens [1,2]. Through the use of small handheld devices that heat and aerosolize liquids into ultrafine particles, vape users inhale nicotine and other noxious compounds deep into the lungs [3,4]. Since 2019, there have been increasing reports of vape-induced pulmonary disease with some severe enough to cause death [3]. Of note, vape cartridges containing tetrahydrocannabinol (THC) oil, the psychoactive, “high” producing compound found in marijuana, is frequently used by E-cigarette users [3]. THC-containing E-cigarettes were linked to lung injury through laboratory data findings of Vitamin E acetate [5]. As an additive in THC vape cartridges, vitamin E acetate was present in the bronchoalveolar lavage (BAL) fluid of patients with vape associated pulmonary injury (VAPI) [5].

VAPI is a relatively new diagnosis and was the subject of intense investigation by the Centers for Disease Control and Prevention (CDC) in the Fall of 2019. A diagnosis of exclusion, VAPI, tends to present as an acute or subacute systemic deterioration in young, healthy patients with few known comorbidities [6]. General and non-specific constitutional symptoms may be present, but symptoms can range in presentation and severity. Roughly 97% of patients present with respiratory symptoms such as cough, dyspnea, and chest pain, with 77% also presenting with gastrointestinal symptoms [7]. We report the case of an otherwise healthy young man who presented with malaise, worsening shortness of breath, and anorexia who was found on chest CT to have innumerable pulmonary nodules in a "miliary" pattern.

## Case presentation

A 34-year-old male with no relevant past medical history presented with a two-week history of malaise, fatigue, cough, worsening shortness of breath, and 25-pound weight loss secondary to nausea and diarrhea. He denied any pleuritic chest pain, chest tightness, sputum production, or hemoptysis. One week prior, he was seen by another healthcare provider and diagnosed with bronchitis after undergoing a chest X-ray. He reported completing a three-day course of once-daily azithromycin 500 mg tablets, which provided no symptom relief. He denied body aches but reported a “weird” feeling in his chest. Upon further questioning, he denied any recent international travel, recent sick contacts, or high-risk behaviors. He reported working a corporate job and had not traveled more than 10 miles from his home each day for the past three months. 

**Figure 1:**
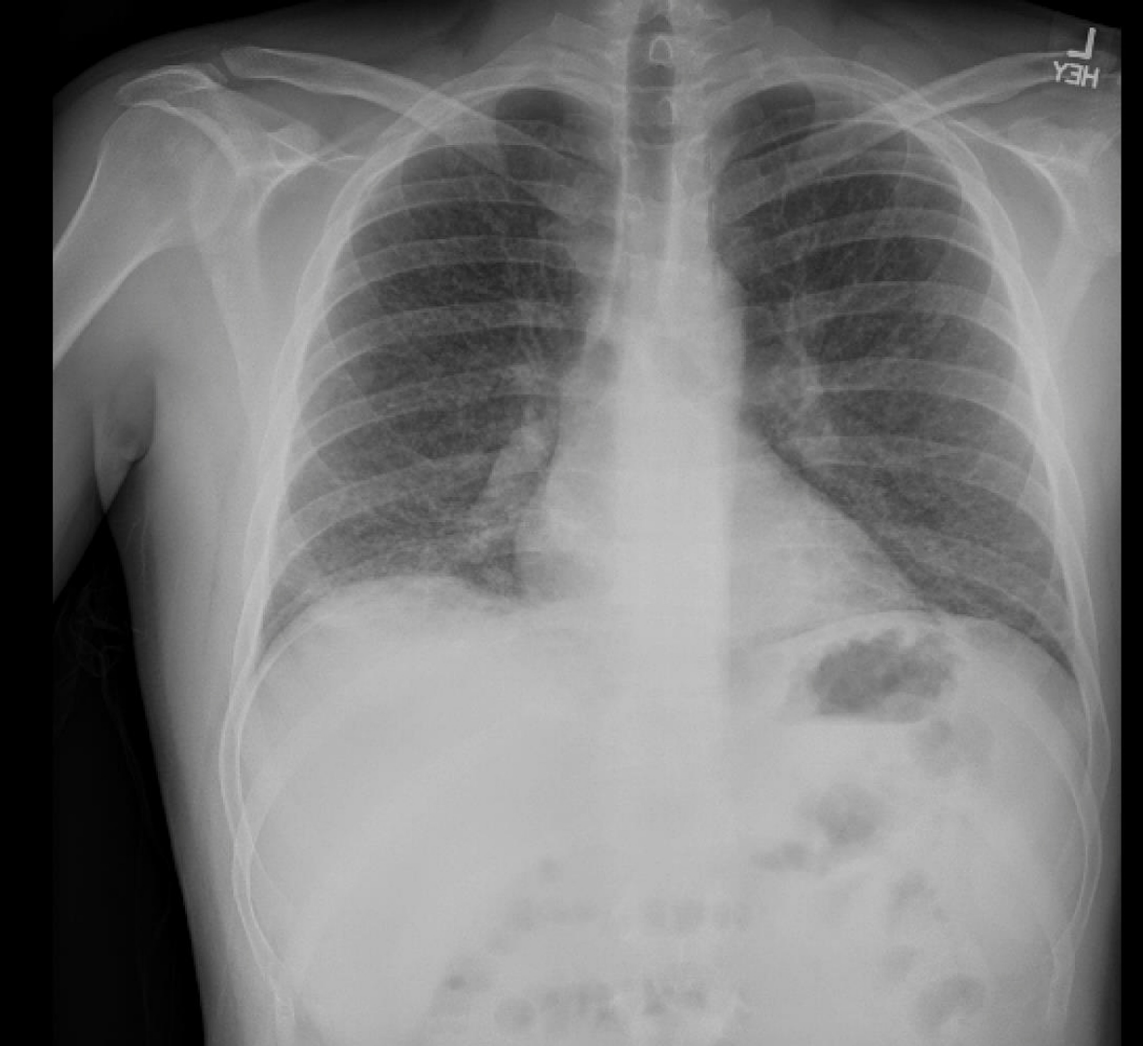
Chest X-ray in the emergency department. Image shows increased interstitial markings with suggestion of a right lower lobe more focal consolidation.

**Figure 2:**
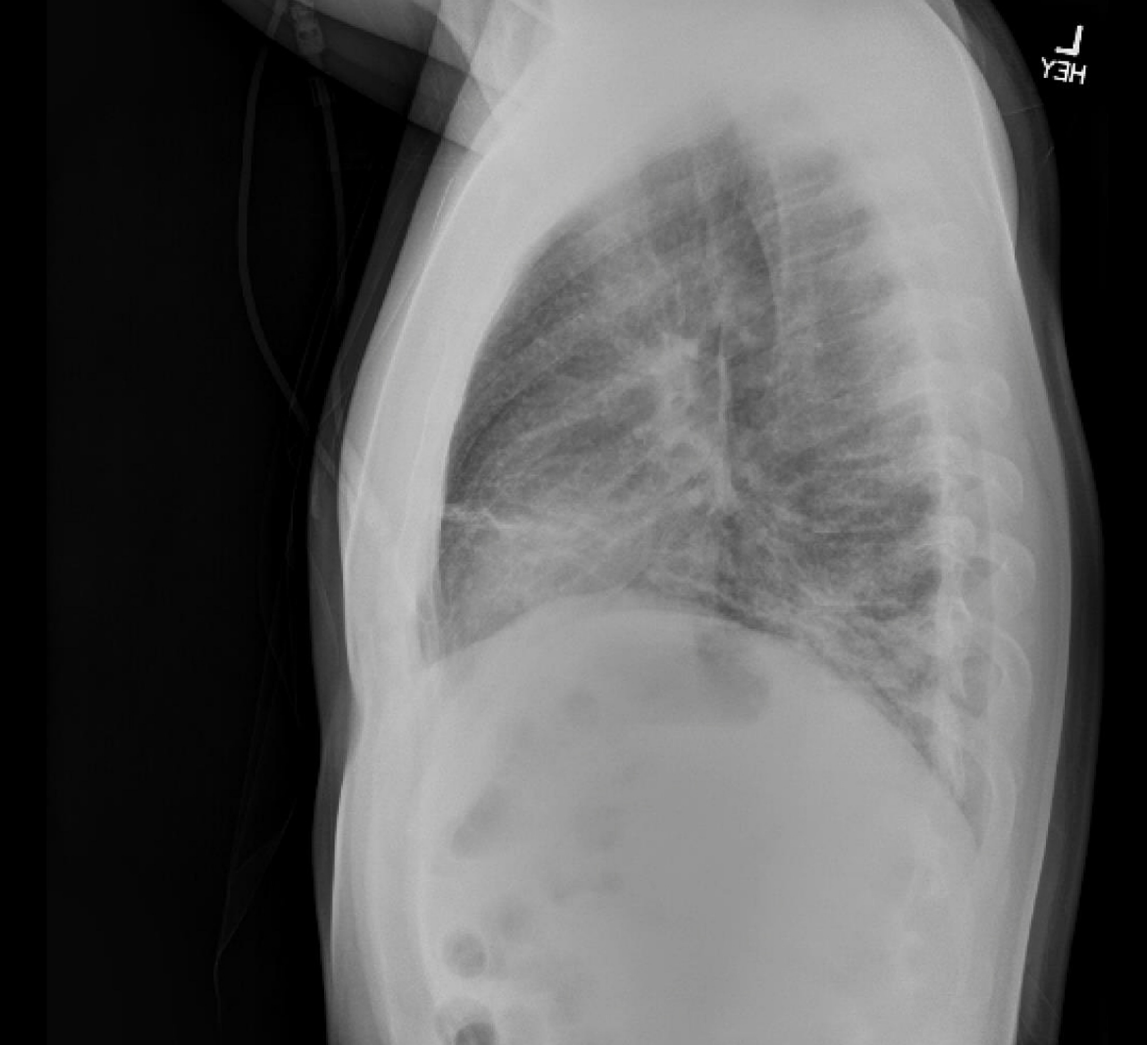
Chest X-ray in the emergency department (lateral view). Image shows increased interstitial markings with suggestion of a right lower lobe more focal consolidation.

**Figure 3:**
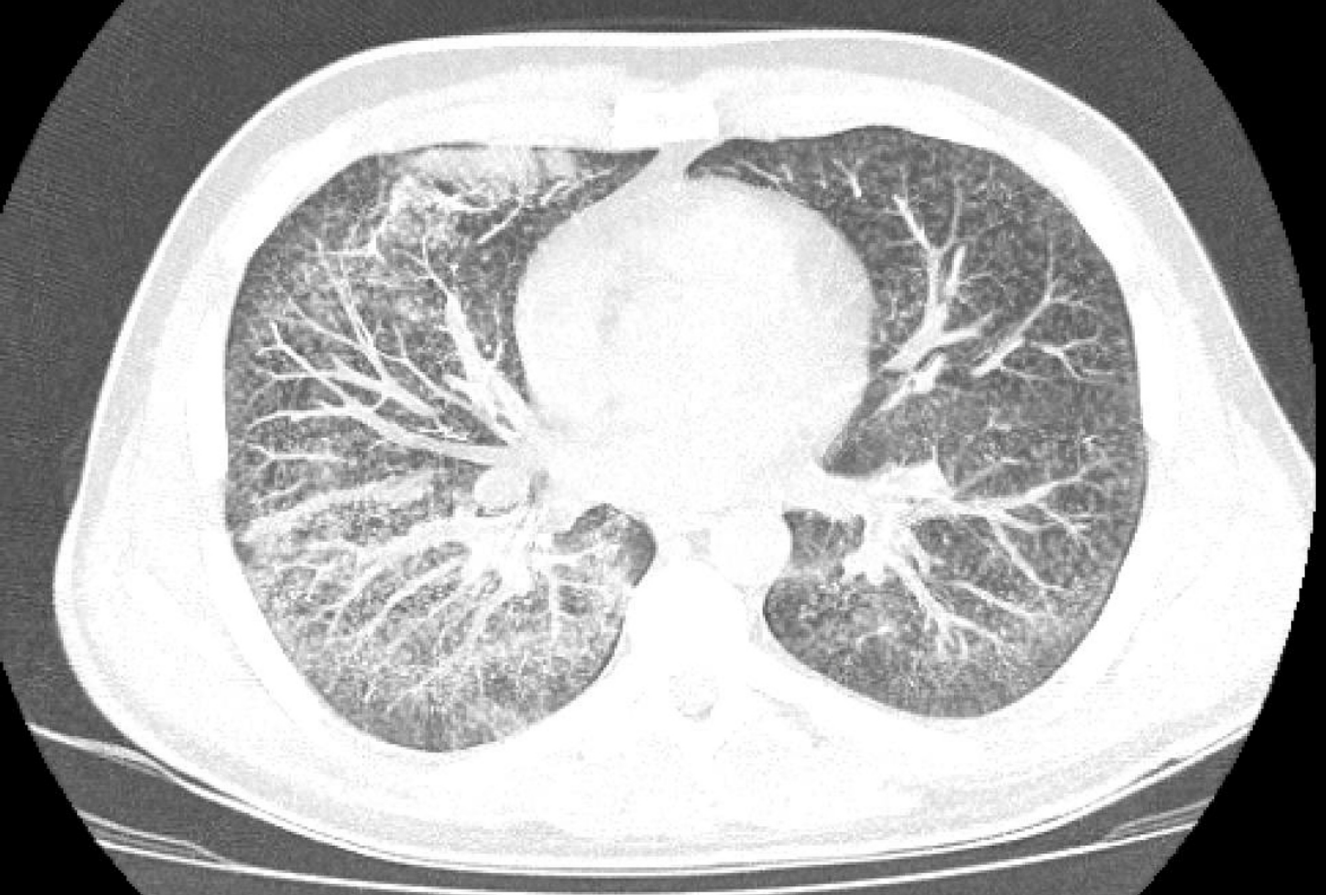
Chest CT on admission. Image shows scattered pulmonary micronodules seen in a predominantly random miliary pattern with bibasilar patchy opacities.

In the emergency department, he was ill-appearing, tachycardiac, and tachypneic with diminished breath sounds toward the lower lobes bilaterally. Oxygen saturations ranged from 89%-95% on room air. He was placed on two to six liters of oxygen delivered via nasal cannula, and oxygen saturations improved to greater than 96% at rest. Initial laboratory workup was significant for C-reactive protein of 386 mg/L, AST of 101 IU/L, ALT of 86 IU/L, white blood cell count of 10.7 thou/cu mm (neutrophil predominant) with a platelet count of 478 thou/cu mm, and an INR of 1.8. A chest X-ray was significant for increased interstitial markings with a few air bronchograms suggesting right lower lobe focal consolidation (Figures 1, 2). A subsequent non-contrast chest CT scan showed numerous miliary pulmonary nodules in a random pattern with bibasilar patchy consolidations (Figure 3). Given the increased suspicion for miliary tuberculosis, the patient was placed in isolation and under respiratory precautions. Infectious disease and pulmonology were consulted to assist in further workup and management.

Further testing, including respiratory and GI polymerase chain reaction (PCR) panels, HIV, hepatitis panel, QuantiFERON, and blood cultures, were unrevealing. As malignancy with pulmonary metastasis was also a diagnostic consideration, tumor markers and a non-contrast CT of the abdomen and pelvis were ordered. The CT scans were unrevealing, and all tumor markers were within normal limits. During his hospitalization, the patient revealed his history of THC-vape use for the preceding six months. This new information heightened our clinical suspicion for VAPI, and a flexible bronchoscopy with biopsy was pursued to confirm the diagnosis and rule out other infectious etiologies. Testing for Histoplasma, tularemia, and cryptococcus were performed and returned negative results. Histopathology from the bronchoscopy revealed intra-alveolar granulation tissue consistent with organizing pneumonia, with no evidence of acid-fast organisms, pneumocystis, fungi, or malignancy. Cytology later confirmed the presence of acute and chronic inflammation with lipid-laden macrophages. Systemic corticosteroids were initiated after identifying lipoid pneumonia in conjunction with vape use. Within three days of admission, his transaminitis began to improve, along with his respiratory function with a decreasing need for oxygen support. After five days, the patient was discharged and instructed to follow-up in the pulmonary clinic within a week and advised to discontinue vaping.

## Discussion

To our knowledge, this is the first example of a "miliary" pattern on chest imaging associated with vaping. Before confirming the diagnosis, infectious etiologies such as tuberculosis were much higher on the differential diagnosis, but the patient had no known exposures or risk factors. VAPI has been associated with several radiographic patterns such as diffuse alveolar damage, organizing pneumonia, and hypersensitivity pneumonitis patterns [8]. CT imaging often demonstrates areas of consolidation, ground-glass changes, nodular consolidation, septal thickening, and mosaic attenuation [9]. The diffuse micronodules in our case were initially concerning for tuberculosis or malignancy. The diagnosis of VAPI and lipoid pneumonia were arrived at after carefully ruling out alternative etiologies and confirming the presence of lipid-laden macrophages. His improvement with corticosteroids and the cessation of THC-vape use also support the diagnosis of VAPI in this case. As our clinical understanding of this condition continues to evolve, our case provides value in demonstrating another radiographic pattern associated with the diagnosis.

The mechanism of lung injury in VAPI is still under investigation, but studies have shown that damage to the lung parenchyma is likely linked to a chemical pneumonitis [10]. The aerosolized liquids inhaled via E-cigarettes contain an array of chemical compounds. Nicotine based E-cigarettes, in addition to nicotine, may include propylene glycol, glycerin, polycyclic aromatic hydrocarbons, nitrosamines, volatile organic chemicals, toxic metals, flavoring compounds, and endotoxins [11,12]. Additionally, E-cigarettes can deliver recreational drugs, including THC-based oils, to users via vape cartridges. In this patient’s case, use of a vape cartridge with a reported 90% THC-based oil. The Food and Drug Administration (FDA) has identified and demonstrated that many THC-containing vape products also contain vitamin E acetate [5]. The degree to which vitamin E acetate is the direct cause of lung injury in VAPI is yet to be determined but is believed to be associated with acute lung injury. The CDC have recommended that THC-containing vape products, especially those containing vitamin E acetate, be avoided [5].

## Conclusions

VAPI should be considered in a patient with a history of E-cigarette use, who presents with pulmonary symptoms and bilateral infiltrates on imaging. CT imaging may be useful in highlighting specific patterns of inflammation that are associated with VAPI. Our case adds a micronodular or "miliary" pattern of nodules to the list of potential radiographic patterns associated with this condition. The patient in our case had a brisk recovery with corticosteroids and cessation of vaping.
